# Association between size at birth, rapid weight gain in infancy, and overweight status among Palestinian refugees under 5 years old: a retrospective cohort study

**DOI:** 10.1016/j.ajcnut.2025.101132

**Published:** 2025-12-04

**Authors:** Zeina Jamaluddine, Oona MR Campbell, Miho Sato, Akihiro Seita, Eric O Ohuma, Edward A Frongillo, Hala Ghattas

**Affiliations:** 1 Department of Infectious Disease Epidemiology and International Health, Faculty of Epidemiology and Population Health, London School of Hygiene and Tropical Medicine, London, United Kingdom; 2 School of Tropical Medicine and Global Health, Nagasaki University, Nagasaki, Japan; 3 Department of Health, United Nations Relief and Works Agency (UNRWA) for Palestinian Refugees in the Near East, UNRWA Headquarters, Amman, Jordan; 4 Department of Health Promotion, Education, and Behavior, University of South Carolina, Columbia, SC, United States

**Keywords:** size at birth, small for gestational age, rapid weight gain, childhood overweight

## Abstract

**Background:**

Evidence on the association of birth size and rapid weight gain with overweight/obesity is inconsistent. Refugees face unique nutritional challenges that influence weight patterns, yet data remain limited.

**Objectives:**

We examined the association between size at birth, infant feeding, and rapid weight gain in the first year of life, and subsequent overweight/obesity in childhood among Palestinian refugees.

**Methods:**

This retrospective cohort study used data from 388,347 live births from 1 January, 2010 to 31 December, 2020, linked to child growth monitoring data using United Nations Relief and Works Agency electronic-health records. The cohort included children aged 0 to 60 mo from 5 regions: Gaza, Jordan, Lebanon, Syria, and the West Bank. Size at birth was categorized into 9 phenotypes, and rapid weight gain was defined as an increase of 0.67 in weight-for-age *z*-score from birth until age 12 mo. Overweight/obesity occurrence was defined as a weight-for-height *z*-score >+2 at any time point after 24 to 60 mo of age. Multilevel mixed-effects models and structural equation modeling were used to analyze associations.

**Results:**

Small for gestational age children had lower odds of exclusive human milk feeding [adjusted odds ratio (aOR): 0.83; 95% confidence interval (CI): 0.81, 0.85], higher odds of rapid weight gain (aOR: 2.39; 95% CI: 2.32, 2.45), and lower odds of overweight/obesity (aOR: 0.50; 95% CI: 0.46, 0.56) compared with appropriate for gestational age children. Large for gestational age children showed lower odds of rapid weight gain (aOR: 0.35; 95% CI: 0.33, 0.36) but higher odds of overweight/obesity (aOR: 2.76; 95% CI: 2.59, 2.95) compared with appropriate for gestational age. Between 22.2% and 34.4% of children experienced rapid weight gain. Rapid weight gain was strongly associated with overweight/obesity at 24 to 60 mo (aOR: 6.53; 95% CI: 6.15, 6.94). Exclusive human milk feeding was associated with lower odds of rapid weight gain (aOR: 0.66; 95% CI: 0.65, 0.67).

**Conclusions:**

Rapid weight gain in infancy, regardless of birth size, is a predictor of childhood overweight/obesity. Exclusive human milk feeding mitigates the risk of rapid weight gain and subsequent overweight/obesity.

## Introduction

To address the rising global prevalence of childhood overweight/obesity, it is essential to understand the mechanisms contributing to overweight/obesity during the critical “first 1000 d” of a child’s life [1,2]. A large body of evidence finds that perinatal influences (including in utero shocks), small size at birth, and early life growth patterns may determine health and developmental outcomes in childhood and later in adulthood [1,[3], [4], [5]].

Small size at birth (including low birthweight, small for gestational age (SGA), and prematurity) is associated with undernutrition (wasting and stunting) during the first 5 y of life [1,6]. However, evidence on the association between small size at birth and childhood overweight/obesity is inconclusive [1,[7], [8], [9], [10]]. An umbrella review identified 8 meta-analyses of the association between small size at birth on overweight/obesity in childhood [1,[7], [8], [9], [10], [11]]. Three of these (2 using low birthweight and 1 using term-SGA as the exposure) found no association [[7], [8], [9], [10], [11]]. One of the 8 meta-analyses (using preterm birth as the exposure) showed an increased relative risk, and 4 of 8 (using low birthweight as the exposure) showed a reduced relative risk. The inconsistencies in these associations may stem from differences in defining the exposure, since low birthweight encompasses both preterm and SGA children. However, the risk factors for, and mechanisms leading to, preterm birth and SGA are different and reflect distinct intrauterine growth conditions, which could result in differential growth patterns postnatally.

Following birth, infant feeding practices influence growth patterns. Exclusive human milk feeding during the first 6 mo has been proposed as protective against childhood overweight/obesity [12], potentially through bioactive components regulating appetite and metabolism. However, studies examining feeding practices in relation to birth size and subsequent obesity are limited.

It has been proposed that children born small relative to their gestational age or preterm may experience accelerated postnatal growth in response to intrauterine nutrient deprivation, and that this rapid postnatal growth can lead to childhood obesity [[13], [14], [15], [16], [17]]. However, discussions on rapid weight gain often fail to distinguish between the expected catch-up growth in premature children needed to achieve developmental milestones that have been delayed in utero and accelerated growth in term children, which might indicate excessive energy intake or suboptimal feeding practices [18,19]. Some studies show that children experiencing rapid weight gain during infancy-particularly those born low birthweight, have a higher likelihood of developing overweight or obesity later in life [20]. Rapid weight gain has been further associated with increased abdominal adiposity [21] and heightened insulin resistance, predisposing these individuals to metabolic syndrome and related conditions [[15], [16], [17],^20^,^22^]. Not all low birthweight children undergo rapid weight gain; some maintain lower growth trajectories throughout childhood or might be genetically smaller, which might confer different health risks or benefits. Few studies distinguish between low birthweight associated with prematurity and that occurring in term infants, which might explain heterogeneity in growth rates after birth [20,23]. The timing of weight gain also appears critical. Evidence suggests that excessive weight gain during infancy has a more pronounced impact on long-term fat accumulation and metabolic health compared with weight gain occurring later in childhood [14,23]. This highlights infancy as a sensitive period for shaping future health outcomes through growth and weight patterns.

A systematic review focusing on the effect of rapid weight gain underscores the need for further research on SGA/large for gestational age (LGA) individuals and on the trajectory of rapid weight gain among those who were SGA/LGA compared with those who were born with an appropriate weight for gestational age (AGA) [23].

Refugee populations face unique vulnerabilities, including food insecurity, limited healthcare access, and displacement-related stress. Protracted refugees may also experience rapid nutritional transitions. Palestinian refugees have experienced substantial dietary shifts toward energy-dense, nutrient-poor foods over recent decades, contributing to rising childhood overweight rates despite persistent undernutrition in some subgroups. Some studies suggest that refugee populations often experience mixed outcomes, such as increased rates of low birthweight and preterm delivery [24]. However, longitudinal data on early growth determinants in humanitarian settings remain scarce.

## Objectives

Previous studies examining the associations between birth size, infant feeding, rapid weight gain, and childhood overweight/obesity have primarily been conducted in high-income settings and have produced inconsistent findings. Key unresolved questions include *1*) how different birth size phenotypes (combining gestational age and size for gestational age) differentially influence postnatal growth trajectories and *2*) whether infant feeding practices modify these associations.

We hypothesized that the association between birth size and childhood overweight/obesity (the main primary outcome) would be mediated by exclusive human milk feeding and rapid weight gain during infancy. Specifically, we aimed to investigate associations between 9 size at birth and gestational age phenotypes and: *1*) exclusive human milk feeding during the first 6 mo, *2*) rapid weight gain in the first 12 mo, and *3*) occurrence of overweight/obesity in children aged 24 to 60 mo among Palestinian refugee children.

## Methods

### Population/data source

We used a Palestinian refugee birth cohort, generated from the electronic-health records (e-health system) of the United Nations Relief and Works Agency (UNRWA) [25]. UNRWA is the key provider of free primary healthcare services to Palestinian refugees through 140 clinics in 5 operational settings: Gaza, Jordan, Lebanon, Syria, and the West Bank. The full dataset used encompasses live births from 1 January, 2010 to 31 December, 2020, derived from linking maternal obstetric records to child growth monitoring records for children aged 0 to 60 mo. We restricted the dataset to singleton live births with ≥1 observation after 24 mo. Records were extracted on 14 September, 2021, making this the last date growth monitoring could be recorded. Birth outcomes, including date of birth, gestational age in weeks (based on last menstrual period calculated in the system), and birth weight in grams from hospital records, were recorded in the obstetric history module by nurses or midwives.

The growth monitoring data, including date of the visit, weight in kilograms (kg), length/height in centimeters (cm), head circumference in cm, and feeding practices (as a multiple-choice response including breastmilk, infant formula, complementary feeding) were routinely collected and recorded by trained UNRWA nurses. Potential confounders, including maternal BMI (in kg/m^2^), smoking during pregnancy, household socioeconomic status beyond maternal education, dietary diversity after 6 mo, and physical activity levels, were not available in the administrative health records.

### Study variables

#### Size at birth

Gestational age categories were defined as preterm (22 + 0 to 36 + 6 wk), term (37 + 0 to 41 + 6 wk), and post-term (42 + 0 to 44 + 6 wk). We used gestational age, birthweight, and sex to assign size at birth as SGA (<10th percentile), AGA (between the 10th to 90th percentiles), and LGA (>90th percentile) according to International Fetal and Newborn Growth Consortium for the 21st Century (INTERGROWTH-21st) standards [[26], [27], [28], [29], [30], [31], [32]]. The 9 resulting newborn gestational age and size phenotypes were defined as preterm-SGA, preterm-AGA, preterm-LGA, term-SGA, term-AGA, term-LGA, post-term-SGA, post-term-AGA, and post-term-LGA.

#### Exclusive human milk feeding

Exclusive human milk feeding was defined as feeding human milk without any infant formula or other foods for the first 6 mo of life.

#### Anthropometry

Weight-for-age *z*-scores (WAZ) and weight-for-height *z*-scores (WHZ) were derived using age- and sex-specific WHO growth standards [33]. Extreme *z*-scores, defined as those >+5 or <‒5 *z*-scores, were excluded from the analysis. For preterm children (gestational age <37), we applied INTERGROWTH-21st standards for WAZ and WHZ to generate *z*-scores ≤64 wk after conception [or 24 wk after full term (40 wk of gestation)] [34]. After 64 wk, we shifted to using the WHO growth standards. Using preterm-specific charts was necessary because preterm children have different growth patterns compared with term children, and using WHO standards from birth for preterm children would misclassify their growth status during early life compared with term children.

Rapid weight gain was defined as a change in WAZ scores of >0.67 *z*-score from birth until 12 mo (difference between 2 *z*-scores), indicating a crossing of major percentile line in growth standard (2nd, 9th, 25th, 50th, 75th, 91st, and 98th percentile line) [14,35]. In a sensitivity analysis, rapid weight gain was also examined for a period starting after 40 wk post-conception until 12 mo of age (52 wk post-birth). This was conducted to account for potential differences in growth patterns between preterm and full-term children during the early postnatal period.

The primary outcome was the occurrence of overweight/obesity (WHZ > +2) at any time point between 24 mo and 60 mo of age. We combined overweight and obesity as a single outcome due to low individual prevalence in this age group, which limited statistical power for separate analyses. Two additional sensitivity analyses were conducted: first, we explored children with repeated overweight/obesity events, defined as a WHZ score exceeding +2 on ≥2 separate occasions after 24 mo of age. Second, the WHO BMI *z*-score [33] was used instead of WHZ to define overweight/obesity.

### Statistical analysis

The analyses were conducted using Stata 18 (StataCorp) and R statistical software. Logistic and multilevel mixed-effects models were applied to assess the association between size at birth, exclusive human milk feeding, and rapid weight gain with overweight/obesity, adjusting for setting and child age in months. We first examined size at birth (SGA/AGA/LGA) stratified by gestational age categories and adjusted for child age and setting (model 1). We then examined the 9 size at birth phenotypes as the exposure variable (model 2). Finally, we employed generalized structural equation modeling to estimate direct and indirect paths for a recursive path model hypothesized to connect size at birth (9 phenotypes: preterm-SGA, preterm-AGA, preterm-LGA, term-SGA, term-LGA, post-term-SGA, post-term-AGA, post-term-LGA compared with the reference category term-AGA), exclusive human milk feeding for the first 6 mo (yes/no), rapid weight gain within the first 12 mo (yes/no), and overweight/obesity between 24 mo and 60 mo (yes/no), adjusting for setting and child age in months. We assessed the direct, indirect, and total effects of the following pathways simultaneously. We examined 4 pathways linking size at birth to childhood overweight/obesity. The first pathway was the direct association between size at birth and overweight/obesity at 24 to 60 mo. The second pathway examined whether exclusive human milk feeding during the first 6 mo mediated the association between size at birth and overweight/obesity at 24 to 60 months. The third pathway explored whether rapid weight gain in the first 12 mo mediated the association between size at birth and overweight/obesity. The fourth pathway tested a sequential mediation model where size at birth influenced exclusive human milk feeding during 0 to 6 mo, which in turn affected rapid weight gain at 12 mo, ultimately leading to overweight/obesity at 24 to 60 mo.

In order to account for missing data in human milk feeding and rapid weight gain variables, we conducted multiple imputation by chained equations as a sensitivity analysis. Imputation was performed using logistic regression models for the binary variables, with 5 imputed datasets and 10 burn-in iterations to ensure convergence of the imputation model. Multiple imputation by chained equations iteratively imputes missing values for each variable, conditional on observed and imputed values of other variables, incorporating relevant covariates, including child sex, phenotype, maternal education, study site, and age at measurement. Bonferroni correction was applied to account for multiple comparisons.

We assessed model assumptions, including appropriate specification, independence of observations, linearity, and normality of random effects, where applicable. For generalized structural equation modeling, we verified correct model structure, suitable distribution and link functions, and absence of multicollinearity. Model fit was evaluated using likelihood-based statistics and residual diagnostics. Missing data were assumed to be missing at random, with imputation model convergence verified through diagnostic checks.

### Ethics statement

The London School of Hygiene and Tropical Medicine, Nagasaki University, and UNRWA’s research review board reference number 25467 granted approval to use de-identified encrypted data.

## Results

### Characteristics of the cohort

A total of 388,347 singleton live births had a record of birthweight and ≥1 measurement of weight and height after 24 mo. These corresponded to 4,183,934 weight and height observations and formed the basis of the analysis. The detailed cohort selection is presented in Supplemental Material 1. Data quality for the singleton live births is detailed in Supplemental Material 2. In general, data quality was high, with limited missing data (total 0.3% for size at birth and 0.1% for weight and height) or implausible data (total 0.02% for size at birth and 0.58% for weight and height). The main missingness was observed in human milk feeding <6 mo (8.4%) and rapid weight gain (18.4%), the latter depending on measurement at 12 mo. When comparing characteristics of participants with complete data to those with missing data, no significant differences were found in relation to feeding status or rapid weight gain measurement, including sex and mean age of participants.

**FIGURE 1:**
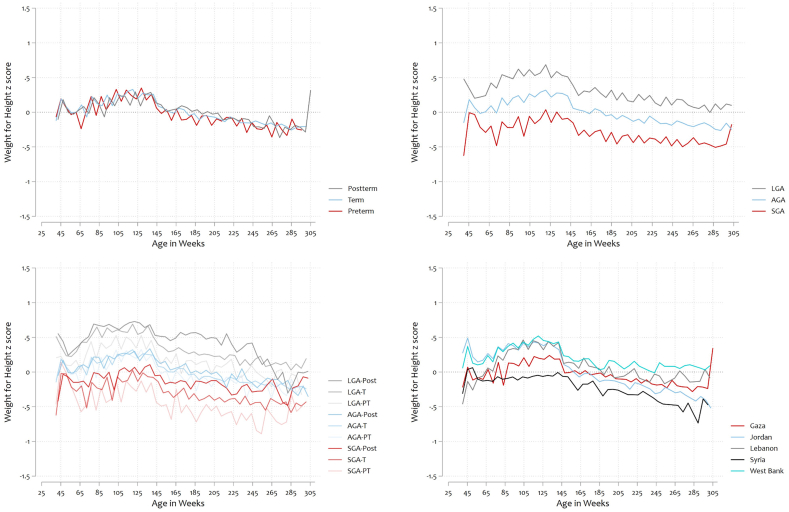
Mean weight-for-height *z*-score by (A) gestational age (B) size for gestational age (C) phenotype (D) setting. Age in weeks from conception. AGA, appropriate for gestational age; LGA, large for gestational age; SGA, small for gestational age; T , term; PT, preterm; Post, post-term.

SGA percentages ranged from 7.5% in Gaza to 11.2% in Jordan, and LGA percentages from 4.8% in Syria to 12.8% in Gaza. Exclusive human milk feeding ranged from 35.4% in Jordan to 77.5% in Syria (Table 1). Rapid weight gain ranged from 22.2% in Gaza to 34.4% in Lebanon. The percentage of overweight/obesity in children (at least once from 24‒60 mo) ranged from 2.4% in Syria to 6.7% in Lebanon (Table 1). Supplemental Material 3 shows the prevalence of exclusive human milk feeding, rapid weight gain, and overweight stratified by exposure (size at birth and gestational age categories). Figure 1 shows growth curves of WHZ by weeks of age (from conception), by size at birth (9 phenotypes), for the 5 settings.

**TABLE 1:** Characteristics of the study population.

Characteristics	Palestinian refugees from:				
	Gaza	Jordan	Lebanon	Syria	West Bank
*n* = 388,347 live births	221,346	85,551	25,205	12,045	44,200
Number of females, *n*	106,698	41,264	12,220	5810	21,329
Number of males, *n*	114,648	44,287	12,985	6235	22,871
Maternal education, *n*	217,677	80,543	24,023	12,034	41,545
Basic, (%)	19.3	41.2	60.9	64.6	27.8
Secondary, (%)	40.2	41.1	15.0	18.1	38.4
Diploma, (%)	5.7	9.3	8.0	7.3	6.8
University and higher, (%)	34.9	8.4	16.1	10.0	27.0
Size for gestational age, *n*	220,643	84,719	25,091	11,983	43,852
SGA, (%)	7.5	11.2	8.3	9.4	8.6
LGA, (%)	12.8	8.1	11.6	4.8	11.1
Gestational age, *n*	220,998	85,092	25,123	11,991	43,891
Preterm, (%)	6.5	7.4	7.6	4.9	6.1
Post-term, (%)	3.2	1.5	1.2	0.8	1.9
Nine phenotypes, *n*	220,643	84,719	25,091	11,983	43,852
Preterm, (%)					
Preterm-SGA	0.4	0.7	0.5	0.4	0.6
Preterm-AGA	4.5	5.4	5.5	3.5	4.4
Preterm-LGA	1.7	1.4	1.6	1.0	1.2
Term, (%)					
Term-SGA	6.4	10.1	7.5	8.6	7.7
Term-AGA	73.1	74.3	73.8	81.9	74.5
Term-LGA	10.8	6.7	10.0	3.8	9.8
Post-term, (%)					
Post-term-SGA	0.7	0.5	0.3	0.3	0.4
Post-term-AGA	2.2	1.0	0.9	0.4	1.4
Post-term-LGA	0.3	0.1	0.1	0.0	0.1
Exclusively human milk feeding, *n*	205,388	81,784	20,556	8624	39,328
Exclusively human milk feeding at 6 mo, (%)	40.4	35.4	39.6	77.5	55.0
Rapid weight gain from birth to 12 mo, *n*	178,711	78,799	16,693	6903	35,582
Rapid weight gain, (%)	22.2	29.7	34.4	28.0	28.4
Anthropometry 24–60-mo, *n*	221,346	85,551	25,205	12,045	44,200
Measurement of wasting at least once (W/H *z*-score <‒2 SD), (%)	4.6	5.8	5.3	5.0	3.7
Measurement of overweight at least once (W/H *z*-score >+2 to ≤+3 SD), (%)	3.1	4.2	5.6	2.1	5.6
Measurement of obesity at least once (W/H *z*-score >+3 SD), (%)	0.4	0.7	1.7	0.4	1.1
Overweight/obesity at 24–60-mo					
Measurement of overweight/obese (at least once measured), (%)	3.4	4.7	6.7	2.4	6.4
Measurement of overweight/obese (at least twice measured), (%)	2.9	3.6	5.9	1.5	4.9

Abbreviations: AGA, appropriate for gestational age; LGA, large for gestational age; SGA, small for gestational age; W/H, weight/height.

After adjusting for setting and child age, SGA children exhibited 17% lower odds of exclusive human milk feeding [adjusted odds ratio (aOR): 0.83; 95% confidence interval (CI): 0.81, 0.85] as compared with AGA children, whereas LGA children also showed 5% lower odds of exclusive human milk feeding (aOR: 0.95; 95% CI: 0.93, 0.97) (Table 2, model 1). Preterm children had 55% lower odds of exclusive human milk feeding (aOR: 0.45; 95% CI: 0.44, 0.47) as compared with term children. Specifically, preterm-SGA children had 73% lower odds of exclusive human milk feeding (aOR: 0.27; 95% CI: 0.24, 0.30) as compared with AGA term children Table 2, model 1).

**TABLE 2:** The association of size at birth and gestational age with exclusively human milk feeding at 6 mo, rapid weight at 12 mo, and overweight/obesity (24‒59 mo). Models adjusted for setting and child age.

Number children	Exclusive human milk feeding 6 mo		Rapid weight gain at 12 mo		Overweight/obesity 24‒60 mo	
	*n* = 353,763 aOR^1^	95% CI	*n* = 315,932 aOR^1^	95% CI	*n* = 380,406 aOR^2^	95% CI
Model 1						
Size for gestational age						
SGA	0.83	(0.81, 0.85)	2.39	(2.32, 2.45)	0.50	(0.46, 0.56)
AGA (reference)	1.00	–	1.00	–	1.00	–
LGA	0.95	(0.93, 0.97)	0.35	(0.33, 0.36)	2.76	(2.59, 2.95)
Gestational age						
Preterm	0.45	(0.44, 0.47)	1.22	(1.18, 1.26)	1.11	(1.00, 1.21)
Term (reference)	1.00	–	1.00	–	1.00	–
Post-term	1.16	(1.12, 1.22)	0.66	(0.62, 0.70)	1.09	(0.92, 1.28)
Model 2						
Preterm-SGA	0.27	(0.24, 0.30)	4.20	(3.78, 4.65)	0.36	(0.24, 0.55)
Preterm-AGA	0.41	(0.39, 0.42)	1.20	(1.16, 1.25)	1.28	(1.14, 1.42)
Preterm-LGA	0.61	(0.58, 0.65)	0.36	(0.33, 0.40)	2.39	(2.02, 2.83)
Term-SGA	0.82	(0.80, 0.85)	2.35	(2.29, 2.41)	0.51	(0.46, 0.57)
Term-AGA (reference)	1.00	–	1.00	–	1.00	–
Term-LGA	0.90	(0.88, 0.93)	0.35	(0.34, 0.37)	2.85	(2.66, 3.06)
Post-term-SGA	1.13	(1.04, 1.24)	1.39	(1.26, 1.54)	0.74	(0.52, 1.04)
Post-term-AGA	1.09	(1.03, 1.15)	0.70	(0.65, 0.75)	0.89	(0.73, 1.08)
Post-term-LGA	1.14	(0.97, 1.33)	0.22	(0.16, 0.31)	5.58	(3.72, 8.36)

Abbreviations: AGA, appropriate for gestational age; aOR, adjusted odds ratio; CI, confidence interval; LGA, Large for gestational age; SGA, Small for gestational age.

1 Logistic regression adjusted for the setting and child age.

2 Multilevel mixed logistic regression adjusted for the setting, age of the child.

After adjusting for setting and child age, SGA children had 2.39 times the adjusted odds of rapid weight gain (95% CI: 2.32, 2.45) compared with AGA children (Table 2, model 1). Conversely, LGA children displayed lower adjusted odds (aOR: 0.35; 95% CI: 0.33, 0.36). Preterm children had substantially higher odds of rapid weight gain (aOR: 1.22; 95% CI: 1.18, 1.26), compared with term children, whereas post-term children exhibited lower odds (aOR: 0.66; 95% CI: 0.62, 0.70) (Table 2, model 1). Rapid weight gain from 40 weeks of gestation till 12 mo (Supplemental Material 4) shows similar patterns.

SGA children exhibited half the odds of having overweight/obesity (aOR: 0.50; 95%CI: 0.46, 0.56) compared with AGA children after adjusting for setting and child age. In contrast, LGA children displayed substantially higher adjusted odds of having overweight/obesity (aOR: 2.76, 95% CI: 2.59, 2.95) (Table 2, model 1). Examining gestational age categories, neither preterm nor post-term was associated with overweight/obesity as compared with term (Table 2, model 1). Similar results were seen when the outcome of repeated overweight/obesity measures was used (Supplemental Material 4).

### Association of rapid weight gain with overweight/obesity

Rapid weight gain was associated with increased odds of overweight/obesity (aOR: 6.53; 95% CI: 6.15, 6.94) among all children (regardless of size at birth) (Table 3). When stratified by birth size, children born SGA, AGA, and LGA all had varying magnitudes of increased adjusted odds for overweight/obesity associated with rapid weight gain, with LGA children showing the highest adjusted odds at 9.29 (95% CI: 7.98, 10.83) (Table 3). When stratifying by gestational age categories, we found that being born preterm with rapid weight gain was associated with an increased odds of having an overweight/obesity measurement, but that the odds were lower (aOR: 4.45; 95% CI: 3.66, 5.42) than for rapid weight gain overall (aOR: 6.53; 95% CI: 6.15, 6.94) (Table 3).

**TABLE 3:** The association between rapid weight gain with overweight/obesity (24‒60 mo) stratified by size at birth groups and exclusive human milk feeding (presenting each subgroup as a separate model).

Exposure Rapid weight gain	Overall and stratified by	*n* =	Overweight/obesity 24‒60 mo	
			aOR^1^	95% CI
	Total/overall	312,337	6.53	(6.15, 6.94)
Stratified by				
	SGA	25,866	4.52	(3.64, 5.62)
	AGA	248,949	8.36	(7.79, 8.97)
	LGA	36,774	9.29	(7.98, 10.83)
Stratified by				
	Preterm	21,746	4.45	(3.66, 5.42)
	Term	282,938	6.83	(6.41, 7.28)
	Post-term	7653	4.31	(2.90, 6.39)
Stratified by				
	Preterm-SGA	1463	1.69	(0.77, 3.70)
	Preterm-AGA	15,359	6.75	(5.29, 8.61)
	Preterm-LGA	4871	5.83	(3.77, 9.02)
	Term-SGA	22,701	4.96	(3.92, 6.26)
	Term-AGA	228,326	8.57	(7.96, 9.23)
	Term-LGA	31,226	10.13	(8.59, 11.94)
	Post-term-SGA	1675	4.69	(1.76, 12.47)
	Post-term-AGA	5224	5.53	(3.45, 8.85)
	Post-term-LGA	557	4.75	(1.26, 17.88)
Stratified by				
	Not exclusively human milk feeding	189,444	7.01	(6.47, 7.59)
	Exclusively human milk feeding	119,223	5.72	(5.16, 6.35)

Abbreviations: AGA, appropriate for gestational age; aOR, adjusted odds ratio; CI, confidence interval; LGA, large for gestational age; SGA, small for gestational age.

1 Multilevel mixed logistic regression adjusted for the setting, age of the child.

Stratifying by combinations of gestational age and birth weight as risk factors indicates a complex interplay of these factors in shaping the adjusted odds of having overweight/obesity. The results revealed that rapid weight gain was associated with increased odds of overweight/obesity across most categories, with the strongest association observed among term infants born LGA [highest aOR of 10.13 (95% CI: 8.59, 11.94)]. Among term infants, a clear gradient was evident, with the risk increasing from SGA to LGA. For preterm infants, AGA infants had the highest risk (aOR: 6.75; 95% CI: 5.29, 8.61), followed closely by LGA infants. Preterm-SGA infants had the lowest aOR overall, at 1.69 (95% CI: 0.77, 3.70), and this was the only group where the confidence interval included 1, suggesting a potentially non-significant association (Table 3). Post-term infants showed fewer clear associations, with post-term-AGA having the highest aOR in this group at 5.53 (95% CI: 3.45, 8.85) (Table 3). Overall, these findings suggest that rapid weight gain is a significant risk factor for overweight/obesity, particularly in term and preterm infants born AGA or LGA.

The association of rapid weight gain with overweight/obesity varied according to lactation practices, with rapid weight gain associated with overweight/obesity among those not exclusively human milk fed (aOR: 7.01; 95% CI: 6.47, 7.59) and among those exclusively human milk fed (aOR: 5.72; 95% CI: 5.16, 6.35).

When analyzing these data with the BMI *z*-score to define overweight/obesity instead of the WHZ score, we found similar results (Supplemental Material 4).

### Structural equation model

**FIGURE 2:**
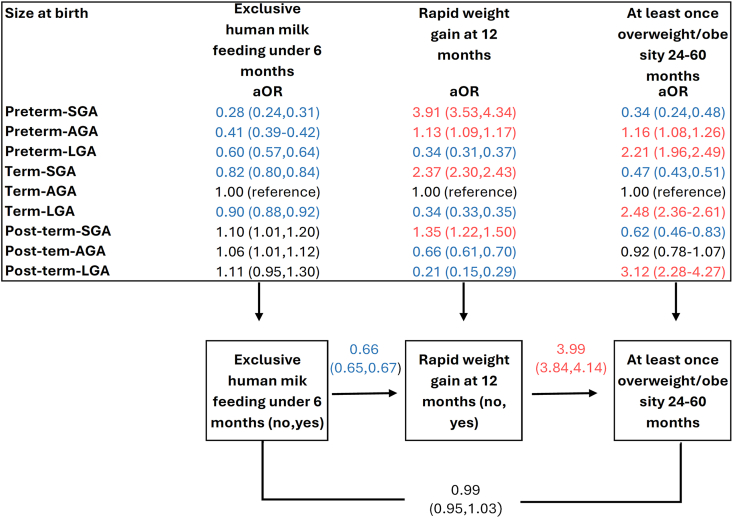
Recursive path model of size at birth, exclusively human milk feeding <6 mo, rapid weight gain at 12 mo, and overweight/obesity (at least once) for children aged 24 to 59 mo, adjusted for setting and child age (*n* = 315,932). This figure displays a generalized structural equation modeling analysis to examine both direct and indirect effects. Direct effects: size at birth and exclusively human milk feeding, rapid weight at 12 mo, and risk of ever having overweight/obese (see top box in the diagram). Indirect effects: size at birth, mediated by exclusive breastfeeding and overweight/obesity, and size at birth, mediated by exclusive human milk feeding, rapid weight gain, and overweight/obesity. Numbers represent adjusted odds ratios (aORs) with 95% confidence intervals. Numbers in red indicate a significantly increased effect. Numbers in blue indicate a significantly decreased effect. The association between post-term-SGA and post-term-LGA with ≥1 episode of overweight becomes non-significant after Bonferroni correction (unadjusted *P* = 0.022) (adjusted *P* = 0.002). AGA, appropriate for gestational age; LGA, large for gestational age; SGA, small for gestational age.

The likelihood of having overweight/obesity differed across different birthweight and gestational age groups, with notable mediation effects through exclusive human milk feeding and rapid weight gain (Figure 2, Supplemental Material 4).

Among preterm and term children, those born SGA had a greater total effect on the odds of having overweight/obesity (preterm aOR: 2.26; 95% CI: 1.39, 3.13/term aOR: 1.54: 95% CI: 1.40, 1.70) largely driven by an indirect effect through rapid weight gain (aOR: 6.69; 95% CI: 5.63, 7.75)/term aOR: 3.30; 95% CI: 3.13, 3.46), despite a substantially lower direct effect (Supplemental Material 4). Preterm-AGA children exhibited an increased total effect (aOR: 1.39; 95% CI: 1.26, 1.56), with modest indirect effects through the combination of exclusive human milk feeding and rapid weight gain (aOR: 1.19; 95% CI: 1.12, 1.27) (Supplemental Material 4). LGA children showed a higher direct effect among preterm, term and post-term on overweight/obesity (preterm aOR: 2.21; 95% CI: 1.94, 2.47/term aOR: 2.48; 95% CI: 2.36, 2.61/post-term aOR: 3.13; 95% CI: 2.15, 4.10) and lower indirect effects through rapid weight gain (preterm aOR: 0.27; 95% CI: 0.20, 0.26/term aOR: 0.23; 95% CI: 0.21, 0.24/post-term aOR: 0.11; 95% CI: 0.05, 0.16)(Supplemental Material 4). This suggests that being born LGA may be more directly related to later overweight/obesity risk, independent of mediating factors like exclusive human milk feeding and rapid weight gain.

In addition, sensitivity analyses using multiple imputations yielded results consistent with complete-case analyses, with minimal changes in odds ratios typically varying by only 0.01 places (Supplemental Material 4).

## Discussion

This study emphasizes the importance of rapid weight gain in infancy, irrespective of size at birth, as being associated with overweight/obesity in childhood. Specifically, our study showed that rapid weight gain is associated with being overweight/obesity in early childhood. Being born LGA is positively associated with overweight/obesity between the ages of 24 to 60 mo, whereas being born SGA decreases the odds of overweight/obesity at the same ages. Rapid weight gain during the first 12 mo of life, either measured at birth or after 40 weeks of gestation, is associated with overweight/obesity at ages 24 to 60 mo, irrespective of birth size. Finally, exclusive human milk feeding may reduce the likelihood of rapid weight gain, thereby decreasing the likelihood of overweight/obesity between 24 mo and 60 mo. Our study provides a nuanced understanding of rapid weight gain and the occurrence of overweight/obesity during early childhood, in contrast to many studies in the literature, which rely primarily on cross-sectional data [36,37], small sample sizes [[38], [39], [40]], or take place in high-income countries [41].

### LGA and rapid weight gain in infancy: main path to childhood overweight/obesity

Rapid weight gain in infancy, regardless of size at birth, operates through distinct pathways leading to overweight/obesity. Children experiencing rapid weight gain in the first year of life had 6.53 times the odds of having overweight/obesity after 24 mo than those who did not experience rapid weight gain. This aligns with a meta-analysis showing an odds ratio of 4.12 (95% CI: 1.83, 9.28 with high heterogeneity I^2^ = 89.5%) and adds to the body of literature examining this phenomenon [20]. During early childhood, adipose tissue develops rapidly, and excessive energy intake can increase the number and size of fat cells, leading to excess body fat [42,43]. Rapid weight gain during early childhood can also lead to metabolic dysregulation, including insulin resistance and altered appetite. Children of low birth weight or SGA are more likely to have higher adrenal androgen concentrations, insulin resistance, and central fat deposition, thus heightening vulnerability to weight gain and adiposity [22,[44], [45], [46]]. In our study, we found that SGA children had higher odds of rapid weight gain, which elevated their risk of overweight/obesity.

A recent umbrella review showed that high birth weight was associated with the subsequent risk of having overweight/obesity [1]. Our study further emphasizes that being born LGA, not just high birthweight, is key to the development of overweight/obesity in children. Despite LGA being associated with a reduced likelihood of rapid weight gain, LGA was independently associated with an increased risk of overweight/obesity. Children born LGA face challenges in maintaining balanced body proportions from birth onward, as their weight gain outpaces their gain in height [47]. The combination of LGA and rapid weight gain conferred the highest odds of overweight/obesity, highlighting the importance of addressing this dual risk factor. In contrast, we find that SGA was associated with reduced odds of overweight/obesity.

Our findings also emphasized the positive association of exclusive human milk feeding for ≥6 mo with a lower risk of subsequent overweight/obesity during childhood (24‒60 mo) mediated by having a lower odds of rapid weight gain. Studies suggest that the nutritional composition of human milk, including hormones like leptin and ghrelin involved in appetite and energy balance regulation [48], plays a crucial role in protecting against overweight/obesity. One study noted that formula or mixed-fed infants had higher energy intakes at 4 mo, and this was associated with increased weight gain from birth to age 1, 2, or 3 y, as well as higher BMI at ages 1‒5 y [49].

### Strengths and limitations

The robustness of our results is supported by consistency across all sensitivity analyses, affirming the study’s internal validity. These analyses included examining 1 setting at a time, restricting rapid weight gain to gain after gestational age >40 wk, using BMI *z*-score instead of WHZ score for overweight/obesity classification, and examining repeated overweight/obesity instead of using a single measurement of overweight/obesity after 24 mo.

This study leveraged administrative electronic growth monitoring data collected regularly, distinguishing it from cohorts with less frequent measurements and enabling the assembling of a very large dataset. However, specific intervals between data collection time points vary, and certain variables relevant to the association, such as dietary diversity, were not included. Additionally, the data may have been susceptible to selection bias, as the most vulnerable infants and children might not have attended regular growth monitoring sessions. Since growth monitoring was limited to children <5, it was not possible to examine whether this association persisted among older children, adolescents, or adults. Electronic-health records are subject to information and recall bias, especially when it comes to exclusive human milk feeding. The structural equation model assumed exclusive human milk feeding was a determinant of rapid weight gain, but some evidence in the literature suggests the reverse causality, where rapidly growing children are more likely to be kept exclusively human milk fed, whereas slower-growing children are more likely to have other foods introduced early [50].

To define rapid weight gain, we opted for a WAZ increase of >0.67 from birth to 12 mo, despite the existence of other methods like conditional relative weight gain, which account for previous weight and growth patterns. We chose this method because it is widely used in the pediatric literature and facilitates comparisons with growth charts[20]. Although the WAZ increase of >0.67 has limitations in capturing subtle growth changes, it provides a straightforward and clinically intuitive assessment of rapid weight gain in our diverse study population. Our analysis showed similar results when using WHZ or BMI *z*-score. Both BMI *z*-scores and WHZ have limitations in accurately reflecting body composition, however, particularly in young children. Future studies incorporating direct measurements of fat mass and lean mass would provide more precise assessments of body composition and obesity risk in this age group [51]. Although growth may differ by sex, we adjusted for sex rather than disaggregating the analyses by sex. Future research could explore sex-specific growth patterns in this population.

Although our study focuses on Palestinian refugee children, the biological pathways examined are likely to operate across ethnic groups and contexts. Consistent findings across 5 geographically diverse settings support the generalizability of these associations. The strong link between rapid infant weight gain and later overweight/obesity highlights a critical intervention window in the first year of life. In humanitarian settings like Gaza and Syria, where acute food insecurity alternates with recovery, understanding growth dynamics is vital to promote healthy, rapid weight gain without excessive adiposity. Exclusive human milk feeding shows a protective effect, supporting its promotion as a critical intervention in crisis-affected settings. Elevated risk among LGA infants underscores the need for targeted infant and young child feeding counseling and support for these children starting from birth. Key interventions supporting exclusive human milk feeding for 6 mo (including in conflict-affected settings), and promoting optimal infant feeding practices, particularly for infants at high risk of rapid weight gain, such as SGA and LGA infants.

In conclusion, by addressing gaps in the existing literature, our study advances the understanding of early life determinants influencing childhood overweight/obesity, providing insights for the development of public health strategies focused on prevention and intervention.

## Author contributions

The authors’ responsibilities were as follows– ZJ, OMRC, HG: developed the conceptualization and methodology; ZJ: led the data curation and formal analysis, and was supported by OMRC, HG, EOO, EAF; ZJ, OMRC, HG: primarily conducted the drafting of the manuscript; AS: provided the data; and all authors: read and approved the final manuscript.

## Data availability

Data described in the manuscript, code book, and analytic code will be made available upon request pending from United Nations Relief and Works Agency (data custodian).

## Funding

ZJ was supported by the Nagasaki University “Doctoral Program for World-leading Innovative and Smart Education” for Global Health (KYOIKU KENKYU SHIEN KEIHI), MEXT (Ministry of Education, Culture, Sports, Science and Technology), Japan. The funder had no role in study design, data collection, data analysis, data interpretation, or writing.

## Conflict of interest

Edward A. Frongillo is a member of the Statistical Review Board for The American Journal of Clinical Nutrition and played no role in the Journal's evaluation of the manuscript
