# Supplementary material for: Association between size at birth, rapid weight gain in infancy, and overweight status among Palestinian refugees under 5 years old: a retrospective cohort study
Source: Am J Clin Nutr. 2025 Dec 4;123(2):101132. doi: 10.1016/j.ajcnut.2025.101132 (PMC12917216; doi:10.1016/j.ajcnut.2025.101132)
Supplement: Multimedia component 1 [file mmc1.docx]

**Supplementary materials**

[Supplementary material 1– Characteristic of the linked population 2](#_Toc194268975)

[Supplementary material 2- Data quality 11](#_Toc194268976)

[Supplementary material 2.1- Quality of size at birth data 11](#_Toc194268977)

[Supplementary material 2.2- Quality of growth monitoring data 16](#_Toc194268978)

[Supplementary material 2.3- Quality of covariate data 18](#_Toc194268979)

[Supplementary material 3- Prevalence of exclusive breastfeeding, rapid weight gain and overweight 20](#_Toc194268980)

[Supplementary material 4- Sensitivity analysis 21](#_Toc194268981)

# **Supplementary material 1– Characteristic of the linked population**

A total of 709,064 livebirths from among 972,743 in Gaza, Jordan, Lebanon, Syria and West Bank between 01/01/2010 and 31/12/2020 were linked with growth monitoring records up to their last visit before 14/09/2021. Most of the livebirth records (709,064) had at least one growth monitoring visit (87%), with a total of 6,309,046 growth monitoring records. There were no differences in loss to follow up by sex or maternal education using Kaplan Meier curves and Cox regression (details below).

Total livebirth records from the mother obstetric records were linked to growth monitoring records of children. Syria did not collect growth monitoring until 2011. The highest extent of linkage between livebirths and growth monitoring records was observed in Gaza (92%), while the lowest was in Syria (78% partly because growth monitoring began later). The extent of growth monitoring data collected by cohort year and their quality improved (Supplementary Figure S.1.4). The percentage of linkage increased over time as the programme was implemented.

Figure S.1.3 shows that the mean number of growth monitoring visits per child varied across the settings and the cohort years. Figure S.1.3 describes the years and sites of peak performance. Jordan served as the pilot setting for growth monitoring, resulting in higher levels of observation at the beginning of the implementation of the e-health system. Gaza and Lebanon had the highest number of visits. The year 2016 had the highest mean number of visits for the total, reflecting the improvement in measurement practices from the earlier cohorts, while also being the point at which those born in 2010 would have reached 60 months. From 2017 onwards the younger cohorts had fewer visits recorded because they had not yet reached age 60 months, and their growth monitoring data collection was still ongoing.

We investigated the frequency of growth-monitoring visits across various age groups of children, categorized by cohort year, to discern trends in timing of anthropometric measurements. Cox regression was used to characterize children lost-to-follow-up within their respective cohorts, including by sex and maternal education. We excluded multiple live births (twins, triplets etc.,) from our analysis, as we could not accurately determine the distinct birthweight of each newborn when dealing with same-sex multiples (20).

We then focused our analysis on individuals with recording at 24 months onwards. This limited the data available to 388,347 livebirths linked to growth monitoring. We will refer to this dataset of singleton as the “overweight UNRWA analysis” dataset.

*Age lost to follow-up.*

To compare among settings, sex, and maternal education, we present Kaplan Meier curves and Cox regression adjusting for setting, sex and maternal education as potential determinants of age lost to follow-up.

*Settings*. We restricted this analysis to cohort of children who would have reached the age of 24-60 months by 2020, cohorts born in 2010 to 2015. Figure S.1.5 shows that over time data collection improved with median age of last measurement increasing (meaning data is collected at even more delayed ages). The clearest trend was observed in Gaza which shows an increase in the median age of last measurement from 2010 to 2015, increasing from 21 till 45 months. The median age of the last growth monitoring visit did not change much from 2010 till 2015 in Jordan, Lebanon, or Syria (Figure 1.5). In the West Bank, the median age of last measurement decreased from 2010 to 2015, from 48 to 36. In 2015 the median age at last measurement was oldest in Lebanon (48 months), followed by Gaza (45 months), West Bank (36 months), Syria (31 months), and Jordan (27 months).

*Education*. Just a very slight difference one (in secondary as compared to basic education) or no difference was observed in the age at last measurement by maternal education.

*Sex*. No difference was observed in the age at last measurement by sex of the child. The hazard ratio for sex was 1.00 (95%CI; 0.99,1.00).

Table S.1.1- Flowchart of data “loss”.

| **Palestinian refugees in** | **Total livebirth records** | **Livebirths records linked with any records of child health outcomes (including growth monitoring)** | **Livebirths excluding multiples (twins/triplets…)** | **Singleton livebirths linked with growth monitoring records** | **Singelton livebirths with a growth monitoring measure at 24 to 60 months** |
| --- | --- | --- | --- | --- | --- |
| Gaza | 438,134 | 411,279 | 406,490 | 377,959 | 221,346 |
| Jordan | 267,624 | 200,271 | 197,295 | 175,612 | 85,551 |
| Lebanon | 56,075 | 49,972 | 48,968 | 41,821 | 25,205 |
| Syria | 64,093 | 43,439 | 42,647 | 30,751 | 12,045 |
| West Bank | 146,817 | 106,910 | 105,155 | 82,921 | 44,200 |
| **Total** | 972,743 | 811,871 | 800,555 | 709,064 | 388,347 |

Figure S1.2. Flowchart
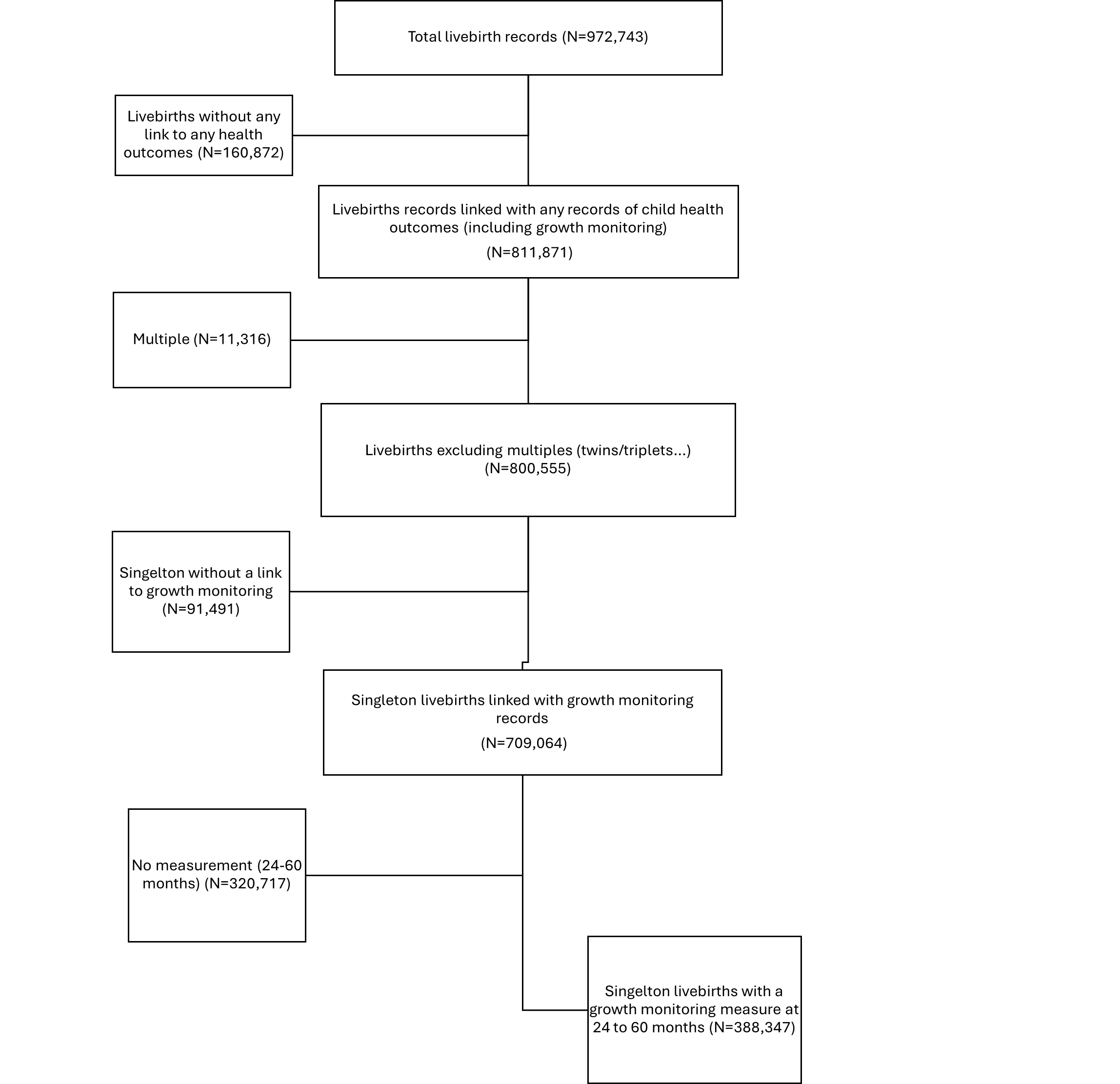


Figure S.1.3- Mean number of growth monitoring visits per child in each setting and cohort.


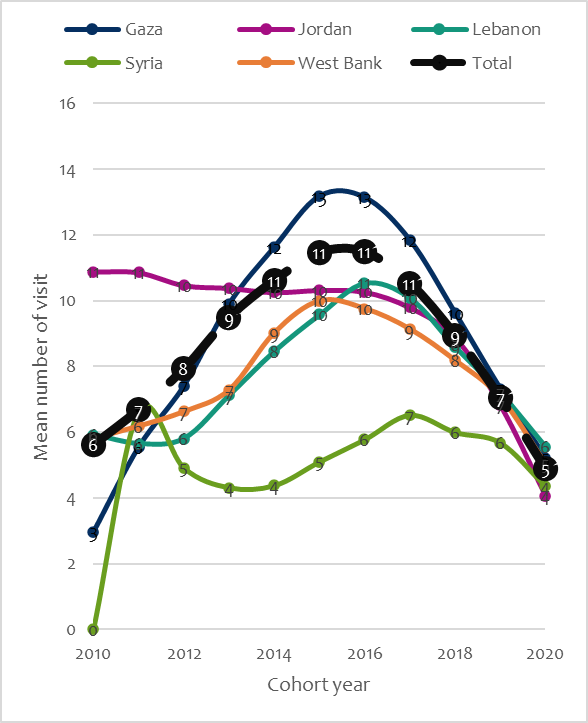


Figure S.1.4- Number of livebirths linked to growth monitoring records by cohort year and by setting in the “overweight UNRWA analysis” dataset.


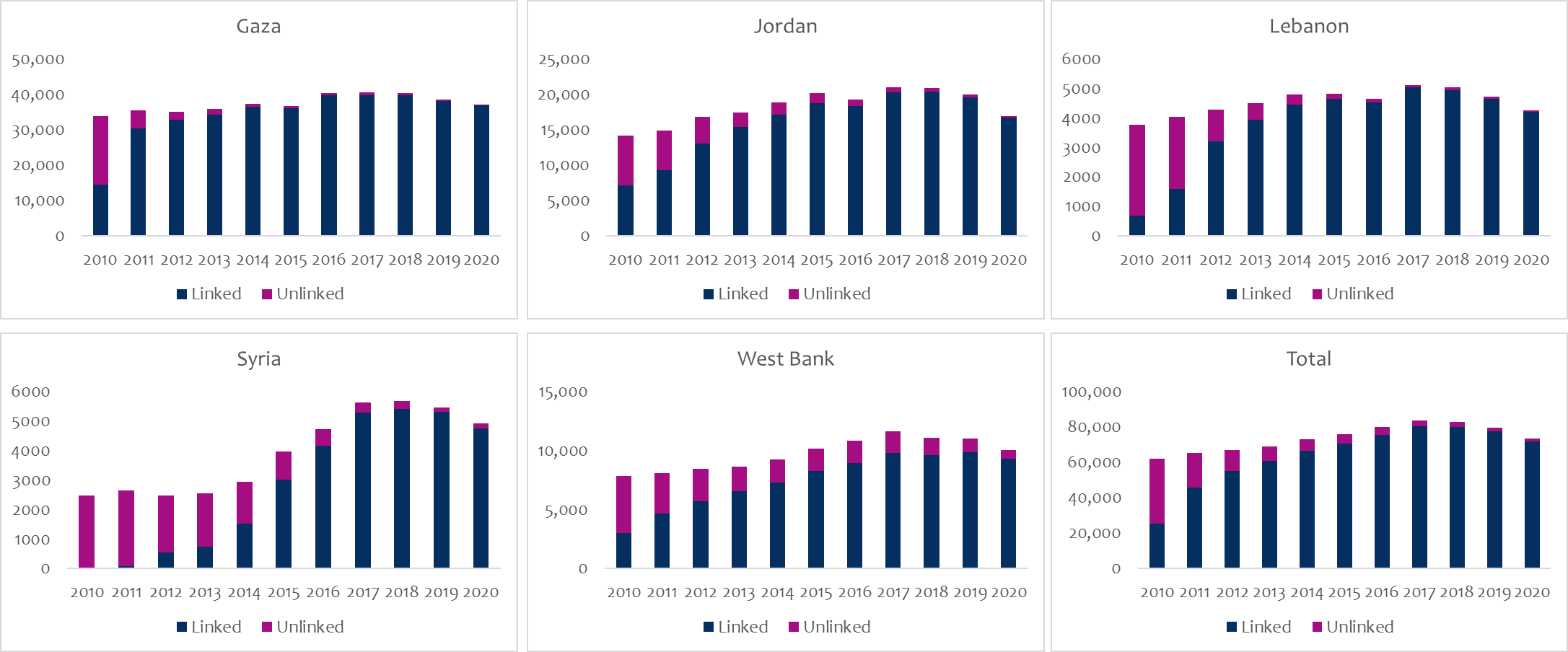


Figure S.1.5- Survival plots of age at last observation stratified by setting and cohorts (2010-2015) for the children reaching the age of 60 months.


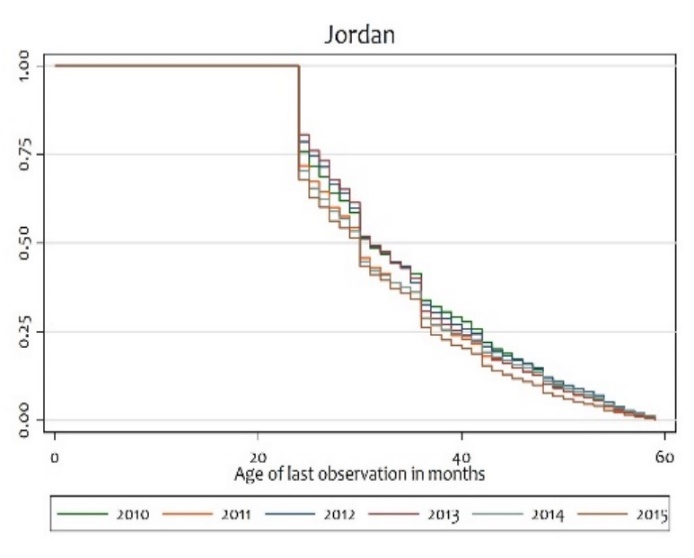

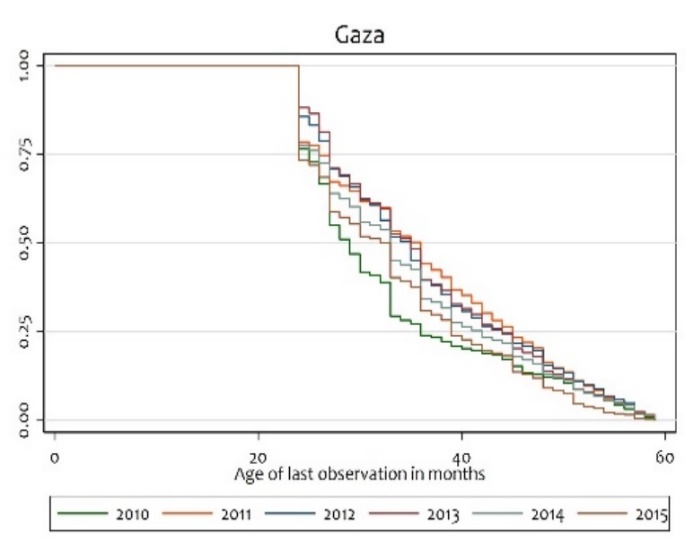

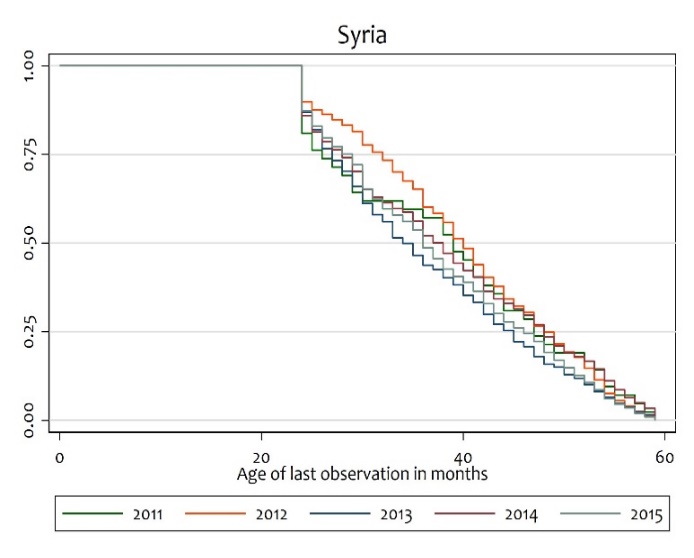

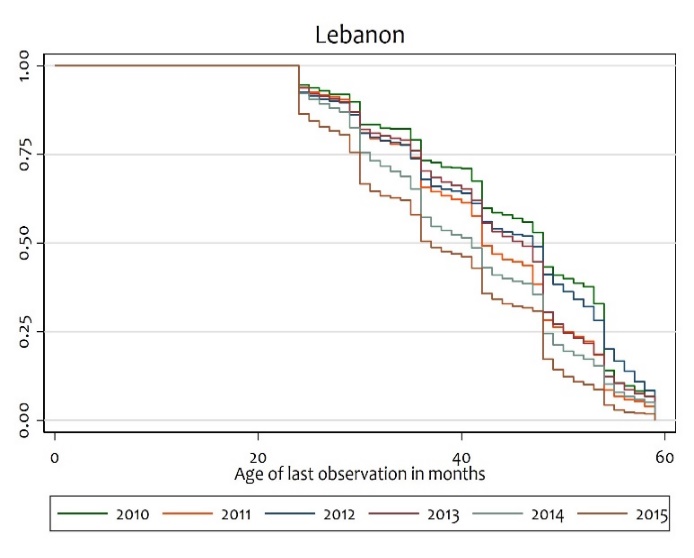

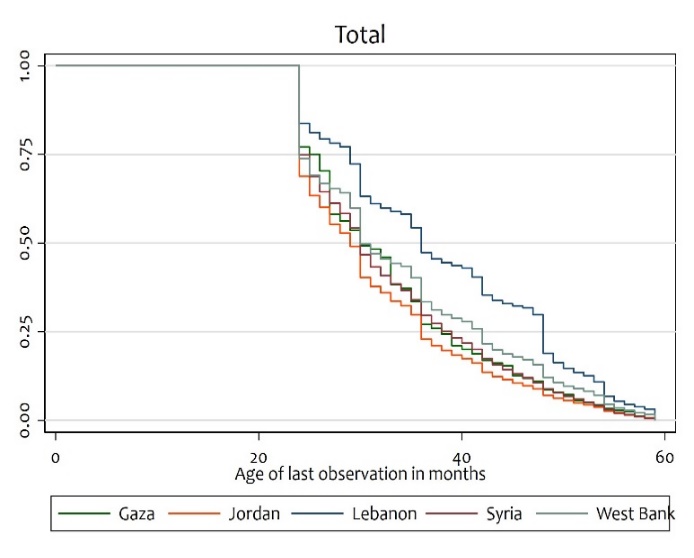

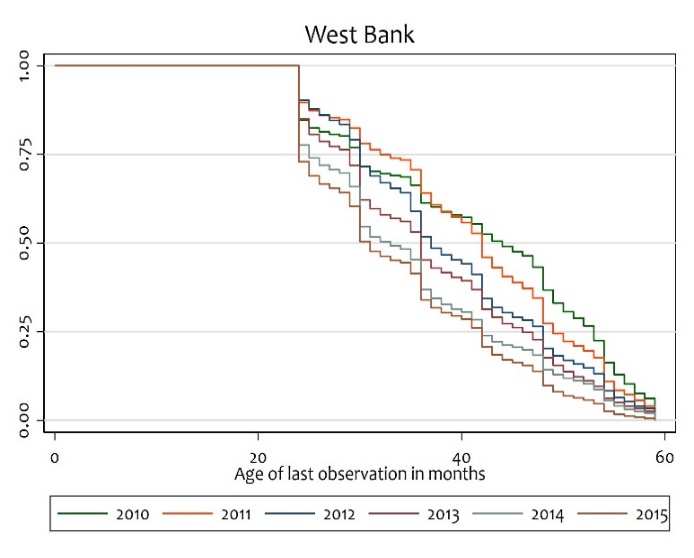


Figure S.1.6- Median age of last observation by setting.


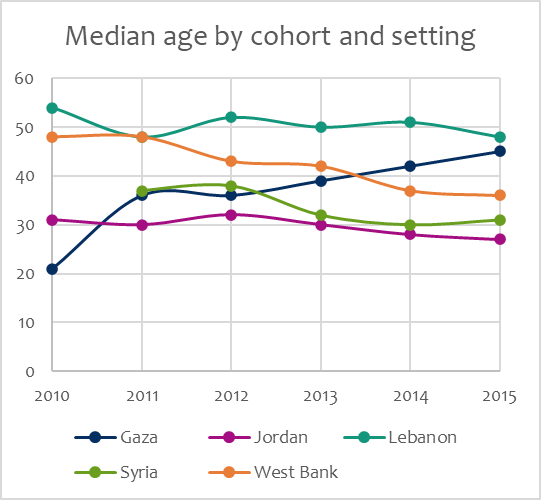


Figure S.1.7- Survival plots of age at last observation stratified by sex and by education cohorts (2010-2015) for the children reaching the age of 60 months.


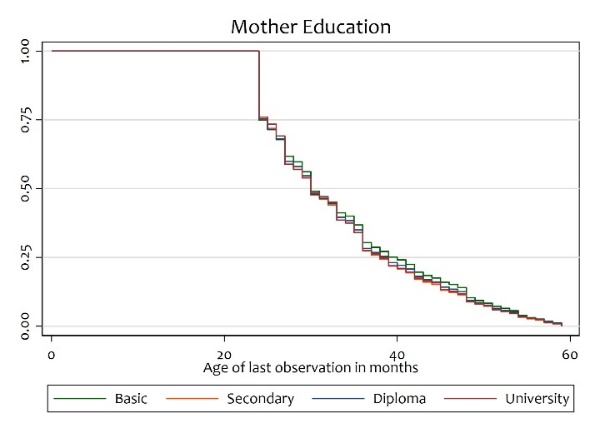

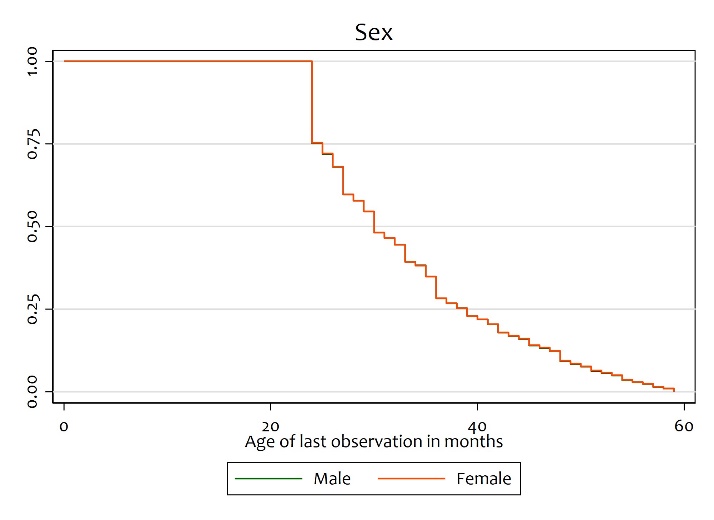


Table S.1.8- Crude association between child sex, mother education setting and last visit in month as outcome using the “overweight UNRWA analysis” dataset.

|  | **Hazard ratio** | 95% CI |
| --- | --- | --- |
| **Child sex** |  |  |
| Male (reference) | 1.00 |  |
| Female | 0.99 | (0.99,1.00) |
| **Mother education** |  |  |
| Basic (reference) | 1.00 |  |
| Secondary | 1.02 | (1.01,1.03) |
| Diploma | 0.99 | (0.98,1.02) |
| University | 1.01 | (1.00,1.02) |
| **Setting** |  |  |
| Gaza (reference) | 1.00 |  |
| Jordan | 1.16 | (1.14, 1.16) |
| Lebanon | 0.63 | (0.62,0.64) |
| Syria | 0.83 | (0.80, 0.86) |
| West Bank | 0.83 | (0.82, 0.84) |

# **Supplementary material 2- Data quality**

We assessed missing data, heaping, digit preference, and range of values for birthweight, gestational age, weight, and height variables. Implausible birthweight, weight and height measurements were excluded. We compared our values to those from population-based surveys in host countries, or associations previously described in the literature (e.g., sex ratio at birth, increasing birthweight with parity).

## **Supplementary material 2.1- Quality of size at birth data**

*Quality of the data*

This section looks at the quality in the analysis dataset, focusing on a cohort of singleton children with at least one measurement of weight and height after 24 months). Out of the 388,347 livebirths 686 had missing birthweight information, 1,247 had missing gestational age data, and 5 had both missing (Table S.2.1.1). Implausible gestational ages beyond 44 weeks (n=121) were also dropped. Implausible or missing data were minimal compromising 0.53% of livebirths (Table S.2.1.1)

When analysing digit preference for birthweight data, we observed that 99% of the data had a zero in the first digit position, 93% in the second digit position, 31% the third digit position. We identified a higher occurrence of heaping at 3,000 grams in the birthweight variable especially in Syria (Figure S.2.1.2). Additionally, we found that there were notable instances of heaping at other specific weights, including 1,000 grams and 500 grams.

We present a two-way scatter plot of birthweight and gestational age for live births to identify implausible data points (Figure S.2.1.3). A consistent and expected pattern is observed when examining the percentage of birthweight classification within different gestational age groups (Figure S.2.1.3). We encountered a few data points that were deemed very unlikely based on gestational age/birthweight.

Overall, the quality of the birthweight and gestational age data is good, with some concern on reporting accuracy (with respect to heaping in measurement).

*External validity*

To further investigate the validity of data from the UNRWA dataset, we compared findings with the most recent literature on LBW and preterm prevalence on the host countries while noting that most comparators do not specify refugee sub-populations. In UNRWA dataset, LBW ranged from 6.19 to 10.5%, aligning with similar percentages reported in Lebanon 12.6% and in the State of Palestine 10.4% (56). Regarding SGA and preterm SGA, our data datasets findings align with prevalence published in the most recent global analysis of SGA prevalence, suggesting a similar pattern to Western Asia and Northern Africa region (Table S.2.1.5).


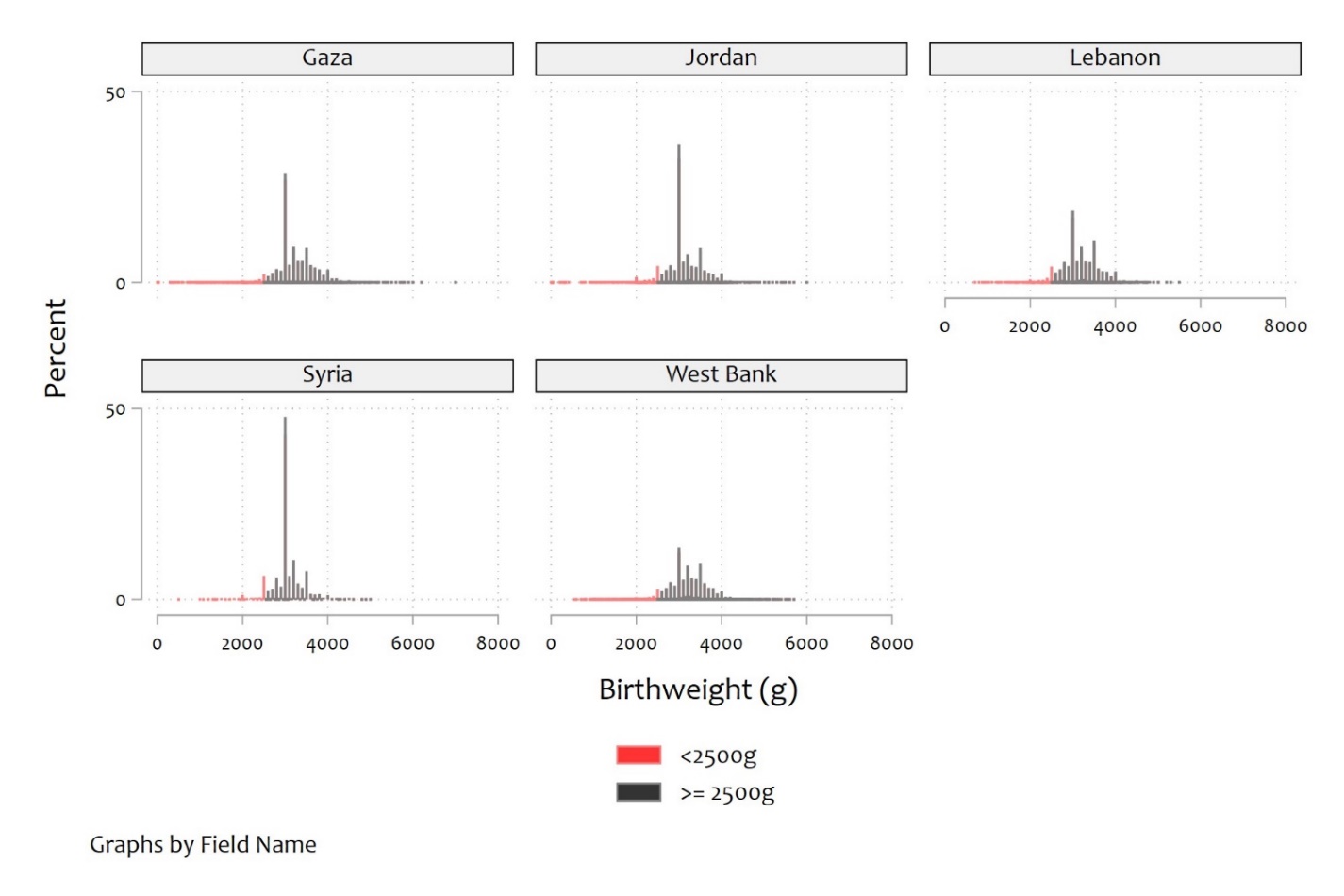
Figure S.2.1.1- Histogram of birthweight by settings using the “overweight UNRWA analysis” dataset.

Table S.2.1.2- Data quality and missing data using the “overweight UNRWA analysis” dataset.

|  | **Gaza** | **Jordan** | **Lebanon** | **Syria** | **West Bank** | **Total** |
| --- | --- | --- | --- | --- | --- | --- |
| Missing birthweight | 257 | 360 | 31 | 5 | 33 | 686 |
| Missing gestational age | 348 | 454 | 82 | 54 | 309 | 1,247 |
| Missing both birthweight and gestational age | 0 | 5 | 0 | 0 | 0 | 5 |
| Gestational age >44 | 98 | 13 | 1 | 3 | 6 | 121 |
| **Total missing** | 703 | 832 | 114 | 62 | 348 | 2,059 |
| **Percent of livebirth missing** | 0.32% | 0.97% | 0.45% | 0.51 % | 0.79% | 0.53% |

Figure S.2.1.3- Scatterplot of birthweight and gestational age by setting using the “overweight UNRWA analysis” dataset.


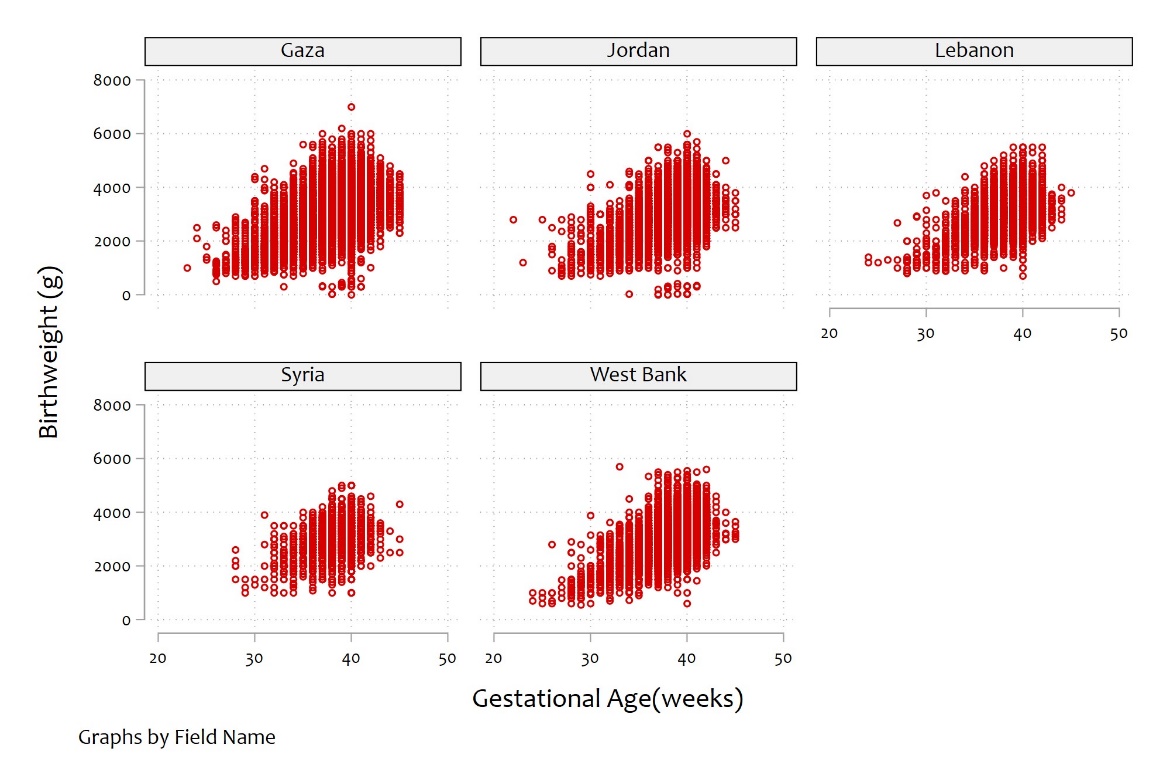


Figure S.2.1.4- Birthweight and gestational age using the “overweight UNRWA analysis” dataset (low birthweight <2500, normal birthweight 2500-4000grams high birthweight >4000).


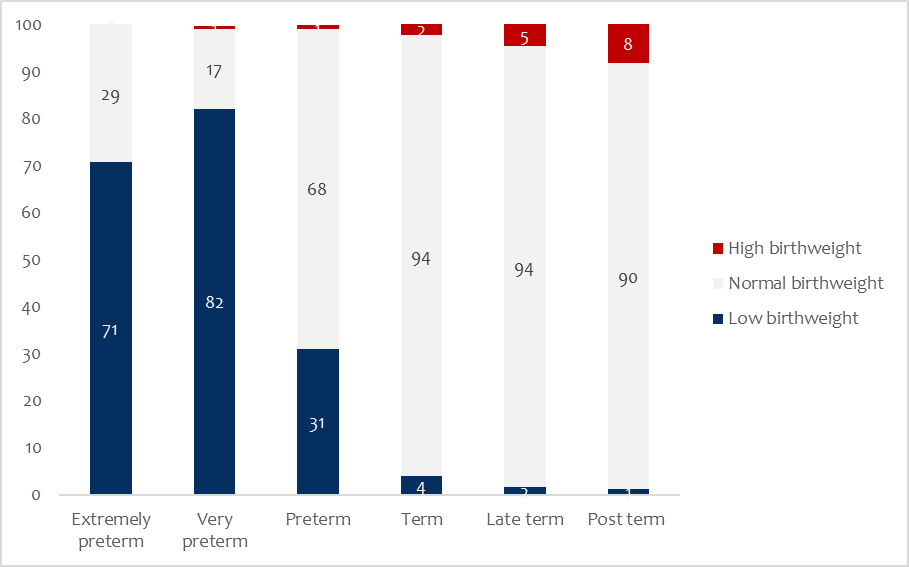


Table S.2.1.5-Percentage of small size at birth in global literature as compared to the Palestinian refugee e-health.

|  | **Preterm SGA** | **Preterm non-AGA LGA** | **Term SGA** |
| --- | --- | --- | --- |
| Palestinian refugees (e-health) | 0.5 | 6.2 | 7.5 |
| Western Asia and Northern Africa (57) | 0.8 (0.6-1.0) | 8.3 (8.0-8.5) | 7.2 (4.3-11.7) |
| Global (57) | 1.1 (0.9-3.1) | 8.8 (6.8-9.0) | 16.3 (14.9-18.9) |

## **Supplementary material 2.2- Quality of growth monitoring data**

*Quality of the data*

UNRWA growth activities use trained personal to measure supine length for children aged up to 23 months and standing height for children aged 24–60 months using infant meters and stadiometers, respectively. The measuring boards used to collect anthropometric measures included ShorrBoards, Seca 217, or locally manufactured boards. Child age was calculated by subtracting the date of interview from the date of birth reported in the questionnaire and was treated as a continuous measure ranging from 0 to 59 completed months (age was floored).

A total of 6,209,913 growth monitoring visits were recorded. We then limited the data to those we used in the analysis based on the cohort definition (at least one measurement after 24 months). Remaining data used for the growth monitoring analysis 4,167,538. We identified missing data in weight and height (0.1%) (Table S.2.2.1). After generating the z-scores, we excluded 24,425 z-scores that were deemed implausible (0.6%) based on WHO guidelines (Table S.2.2.1). As expected, the distributions of HAZ, WAZ, and WHZ followed a normal distribution pattern (data not shown)

*Pattern of data collection*

Figure S.2.2.2 shows the peak age in months when growth monitoring data was collected by the different cohort years. We assume that the data on growth were collected in regular intervals of age during routine monitoring and immunization schedule. The number of growth monitoring visits decreased over time.

Figure S.2.2.2 compiles the peaks observed in the different settings. A total of 33.2% of observations were recorded during these “regular” months, while 66.8% were recorded in “irregular” months. As compared to children who were only measured during the “regular months” the children measured during the irregular period had higher mean number of outpatient visits (data not shown), suggesting that the measurement in “irregular months” may be linked to children who were more “vulnerable” or ill and who might have lost weight leading to fluctuation in weight gain due to sickness. To assess selection bias, we conducted the analysis with and without the measurement of irregular months and find similar results (this is not shown).

Table S.2.2.1- Cleaning of growth monitoring.

|  | **N** | **Percentage of data excluded (%)** |
| --- | --- | --- |
| **Initial N** | **4,183,934** |  |
| Weight equal to 0 | 3,090 | 0.07% |
| Weight missing | 288 | 0.01% |
| Height equal to 0 | 2,526 | 0.06% |
| Height missing | 60 | 0.001% |
|  |  |  |
| Total Excluded weight or height due to missing or equal to 0 | 3,543* | 0.08% |
| Implausible WAZ HAZ WHZ  (HAZ<=-5 or HAZ>=5 or WHZ<=-5 or WHZ>=5 or WAZ<=-5 or WAZ>=5) | 24,425 | 0.58% |
|  |  |  |
| **Total excluded in the analysis** | 27,968 | 0.66% |

*Possibility of overlap

Figure S.2.2.2- Peaks of measurement by age and cohort using the overweight UNRWA analysis” dataset.


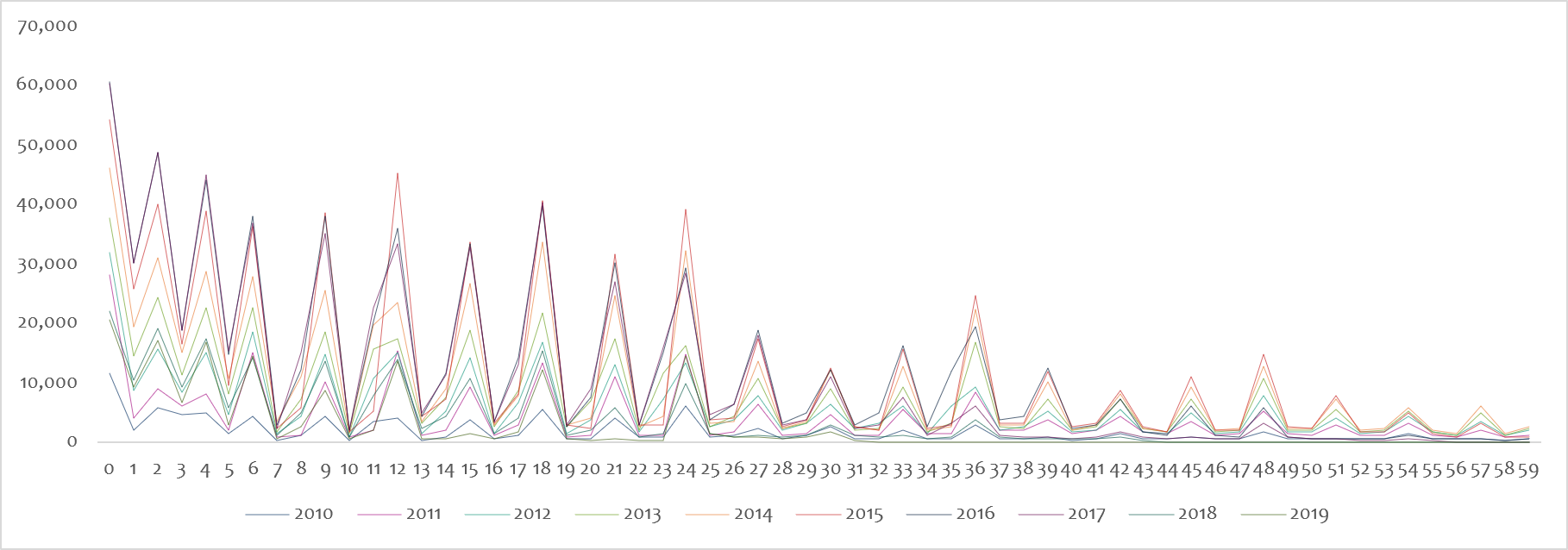


Figure S.2.2.3- Peaks by age and setting

## **Supplementary material 2.3- Quality of covariate data**

The models adjust for sex and maternal education.

*Sex of the child*

The sex ratio ranged from 1.06-1.07 males per female, similar to previously published results from settings where sex-selective abortion is not widespread. The sex ratio is more skewed towards males as compared to the general population (for example Gaza 1.03 in 2022). However, ratio levels remain below 1.07 (levels indicating sex imbalances) (58).

*Maternal education*

The Table below shows the distribution of maternal education by setting of children included in the cohort. The maternal education levels varied across the setting. In Gaza, a significant portion of mothers have completed secondary education (40.2%) or attained university and higher education (34.9%). However, in Lebanon and Syria, a higher proportion of mothers have completed only illiterate/basic/elementary education, with percentages reaching 60.9% and 64.6% respectively. For external validation, when available we compared the prevalence in the dataset with external sources. In general, the proportions in our dataset follow the proportions reported in external reports for Gaza, Lebanon and West Bank (59, 60).

Table S.2.3.1- Maternal education.

| **Palestinian refugees** | **Education level** | **E-health data** | **External reports of** | **Reference** |
| --- | --- | --- | --- | --- |
| Gaza | Illiterate/basic/elementary | 19.25 | 19.0 | (59) |
|  | Diploma | 5.69 |  |  |
|  | Secondary | 40.20 | 35.1 |  |
|  | University and higher | 34.86 | 45.9 |  |
| Jordan | Illiterate/basic/elementary | 41.23 |  |  |
|  | Diploma | 9.33 |  |  |
|  | Secondary | 41.05 |  |  |
|  | University and higher | 8.38 |  |  |
| Lebanon | Illiterate/basic/elementary | 60.9 | 69.9 | (60) |
|  | Diploma | 8.02 | 5.0 |  |
|  | Secondary | 14.98 | 16.6 |  |
|  | University and higher | 16.09 | 8.4 |  |
| Syria | Illiterate/basic/elementary | 64.61 |  |  |
|  | Diploma | 7.30 |  |  |
|  | Secondary | 18.12 |  |  |
|  | University and higher | 9.96 |  |  |
| West Bank | Illiterate/basic/elementary | 27.75 | 19.0 | (59) |
|  | Diploma | 6.82 |  |  |
|  | Secondary | 38.40 | 35.1 |  |
|  | University and higher | 27.03 | 45.9 |  |

# **Supplementary material 3- Prevalence of exclusive breastfeeding, rapid weight gain and overweight**

Table S.3.1 Prevalence of exclusive breastfeeding, rapid weight gain and overweight stratified by exposure (size at birth and gestational age categories)

|  | Exclusive human milk feeding at 6 months | Rapid weight gain | Overweight (1+ measurements) |
| --- | --- | --- | --- |
| SGA | 37.95% | 45.06% | 2.74% |
| AGA | 42.42% | 25.67% | 4.00% |
| LGA | 39.76% | 10.64% | 6.91% |
|  |  |  |  |
| Preterm | 25.26% | 27.42% | 4.94% |
| Term | 42.85% | 25.52% | 4.18% |
| Post term | 45.37% | 20.49% | 3.53% |
|  |  |  |  |

**Table S.3.2 Prevalence of overweight stratified by rapid weight gain**

|  | Overweight (1+ measurements) |  |  |
| --- | --- | --- | --- |
| Rapid weight gain | 8.42% |  |  |
| No rapid weight gain | 2.66% |  |  |

# **Supplementary material 4- Sensitivity analysis**

Table S.4.1 The association between size at birth and gestational age with A) rapid weight gain starting at 40 weeks of gestational age, B) repeated overweight/obesity and C) overweight/obesity as measured using BMI Z score (24 months-60 months).

|  | **A** | | **B** | | **C** | |
| --- | --- | --- | --- | --- | --- | --- |
| Model 1 | **Rapid weight gain at 12 months starting at 40 weeks of gestational age** | | **Repeated overweight/obesity** | | **Overweight/obesity using BMI z score** | |
|  | aOR* | 95% CI | aOR* | 95% CI | aOR* | 95% CI |
| N children | N=285,724 |  | N=386,288 |  | N=379,495 |  |
| SGA | 2.32 | (2.26-2.39) | 0.68 | (0.63-0.74) | 0.58 | (0.53-0.63) |
| AGA (reference) | 1.00 |  | 1.00 |  | 1.00 |  |
| LGA | 0.37 | (0.36-0.39) | 1.73 | (1.85-1.81) | 2.20 | (2.07-2.33) |
|  |  |  |  |  |  |  |
| Preterm | 0.82 | (0.78-0.85) | 1.04 | (0.98-1.12) | 1.11 | (1.03-1.21) |
| Term (reference) | 1.00 |  | 1.00 |  | 1.00 |  |
| Post-term | 0.72 | (0.68-0.77) | 1.10 | (0.97-1.23) | 1.17 | (1.01-1.34) |
| Model 2 | **Rapid weight gain at 12 months** | | **Repeated overweight/obesity** | | **Overweight/obesity using BMI z score** | |
| Preterm-SGA | 2.16 | (1.93-2.41) | 0.62 | (0.45-0.85) | 0.36 | (0.24-0.52) |
| Preterm-AGA | 0.80 | (0.76-0.83) | 1.13 | (1.04-1.23) | 1.27 | (1.16-1.40) |
| Preterm-LGA | 0.32 | (0.30-0.37) | 1.60 | (1.42-1.80) | 2.00 | (1.73-2.32) |
| Term-SGA | 2.31 | (2.25-2.39) | 0.68 | (0.63-0.74) | 0.59 | (0.54-0.64) |
| Term-AGA (reference) | 1.00 |  | 1.00 |  | 1.00 |  |
| Term-LGA | 0.37 | (0.36-0.39) | 1.76 | (1.67-1.85) | 2.26 | (2.12-2.41) |
| Post-term-SGA | 1.55 | (1.39-1.72) | 0.87 | (0.68-1.13) | 0.87 | (0.65-1.17) |
| Post-term-AGA | 0.76 | (0.70-0.81) | 0.97 | (0.84-1.13) | 1.00 | (0.85-1.18) |
| Post-term-LGA | 0.22 | (0.15-0.32) | 2.78 | (2.11-3.69) | 4.13 | (2.87-5.95) |

*aOR= adjusted odds ratio

Table S.4.2 Association between rapid weight gain (measured starting gestational age starting at 40 weeks of gestational age till 12 months) with overweight/obesity (24 months-60 months) stratified by size at birth groups. Multilevel mixed effect logistic regression models adjusted for setting and child age.

|  |  | |  |  | **Overweight/obesity after 24 months to 60 months** | |
| --- | --- | --- | --- | --- | --- | --- |
| Exposure |  |  | | N= | aOR* | 95CI |
| Rapid weight gain starting 40 weeks |  | | Total | 282,421 | 6.14 | (5.7-76.55) |
| Rapid weight gain | Stratified by | | |  |  |  |
|  |  | | SGA | 23,795 | 4.4 | (3.50-5.52) |
|  |  | |  |  |  |  |
|  |  | | AGA | 224,834 | 7.58 | (7.05-8.16) |
|  |  | |  |  |  |  |
|  |  | | LGA | 33,124 | 9.75 | (8.28-11.50) |
| Rapid weight gain | Stratified by | | |  |  |  |
|  |  | | Preterm | 19,126 | 4.04 | (3.24-5.02) |
|  |  | |  |  |  |  |
|  |  | | Term | 255,975 | 6.48 | (6.05-6.93) |
|  |  | |  |  |  |  |
|  |  | | Post-term | 7,320 | 4.44 | (2.93-6.72) |
| Rapid weight gain | Stratified by | | |  |  |  |
|  |  | | Preterm-SGA | 1,293 | 2.44 | (1.06-5.63) |
|  |  | | Preterm-AGA | 13,660 | 5.15 | (3.97-6.67) |
|  |  | | Preterm-LGA | 4,124 | 6.31 | (3.83-10.38) |
|  |  | | Term-SGA | 20,891 | 4.52 | (3.54-5.74) |
|  |  | | Term-AGA | 206,108 | 7.92 | (7.33-8.56) |
|  |  | | Term-LGA | 28,456 | 10.5 | (8.80-12.52) |
|  |  | | Post-term-SGA | 1,675 | 4.69 | (1.76-12.47) |
|  |  | | Post-term-AGA | 5,032 | 5.41 | (3.32-8.82) |
|  |  | | Post-term-LGA | 535 | 7.54 | (1.87-30.26) |
| Rapid weight gain | Stratified by | | |  |  |  |
|  |  | | Not exclusive breastfeeding | 174,512 | 6.32 | (5.82-6.86) |
|  |  | |  |  |  |  |
|  |  | | Exclusive breastfeeding | 119,223 | 5.73 | (5.22-6.31) |

*aOR= adjusted odds ratio

|  | **Overweight/obesity 24-60 months** | | **Overweight/obesity 24-60 months** | | **Overweight/obesity 24-60 months** | | **Overweight/obesity 24-60 months** | |
| --- | --- | --- | --- | --- | --- | --- | --- | --- |
|  | aOR** | 95% CI | aOR** | 95% CI | aOR** | 95% CI | aOR** | 95% CI |
| Size at birth phenotypes |  |  |  |  |  |  |  |  |
| Preterm-SGA | 0.36 | (0.24-0.55) | 0.35 | (0.22-0.53) | 0.20 | (0.13-0.32) | 0.20 | (0.12-0.31) |
| Preterm-AGA | 1.28 | (1.14-1.42) | 1.26 | (1.12-1.41) | 1.19 | (1.06-1.33) | 1.17 | (1.04-1.32) |
| Preterm-LGA | 2.39 | (2.02-2.83) | 2.36 | (1.98-2.82) | 3.72 | (3.10-4.46) | 3.71 | (3.09-4.46) |
| Term-SGA | 0.51 | (0.46-0.57) | 0.50 | (0.45-0.55) | 0.31 | (0.28-0.35) | 0.31 | (0.27-0.35) |
| Term-AGA (reference) | 1.00 | -- | 1.00 | -- | 1.00 | -- | 1.00 | -- |
| Term-LGA | 2.85 | (2.66-3.06) | 2.90 | (2.69-3.12) | 4.44 | (4.10-4.80) | 4.45 | (4.11-4.81) |
| Post-term-SGA | 0.74 | (0.52-1.04) | 0.71 | (0.49-1.02) | 0.60 | (0.41-0.89) | 0.61 | (0.41-0.91) |
| Post-term-AGA | 0.89 | (0.73-1.08) | 0.92 | (0.75-1.13) | 1.07 | (0.87-1.32) | 1.08 | (0.88-1.34) |
| Post-term-LGA | 5.58 | (3.72-8.36) | 5.10 | (3.32-7.83) | 9.94 | (6.38-15.48) | 9.74 | (6.22-15.25) |
|  |  |  |  |  |  |  |  |  |
| Exclusive breastfeeding 6 months |  |  |  |  |  |  |  |  |
| No (reference) |  |  | 1.00 | -- |  |  | 1.00 | -- |
| Yes |  |  | 0.82 | (0.78-0.87) |  |  | 0.92 | (0.87-0.97) |
|  |  |  |  |  |  |  |  |  |
| Rapid weight gain 12 months |  |  |  |  |  |  |  |  |
| No (reference) |  |  |  |  | 1.00 | -- | 1.00 | -- |
| Yes |  |  |  |  | 8.16 | (7.68-8.68) | 8.13 | (7.64-8.65) |

Table S.4.3- The association of size at birth phenotypes and overweight/obesity (BMI Z score) (24-60 months) adjusted for exclusive breastfeeding at 6 months, rapid weight at 12 months, setting and child age.

| **Overweight/obesity** | **Preterm-SGA** | | **Preterm-AGA** | | **Preterm-LGA** | |
| --- | --- | --- | --- | --- | --- | --- |
|  | aOR | 95%CI | aOR | 95%CI | aOR | 95%CI |
| Total effect | 2.26 | (1.39-3.13) | 1.39 | (1.26-1.52) | 0.50 | (0.41-0.59) |
| Direct effect | 0.34 | (0.21-0.46) | 1.16 | (1.07-1.25) | 2.21 | (1.94-2.47) |
| Indirect effect |  |  |  |  |  |  |
| via human milk feeding | 1.00 | (0.96-1.06) | 1.01 | (0.97-1.04) | 1.01 | (0.98-1.02) |
| via human milk feeding rapid weight gain | 6.69 | (5.63-7.75) | 1.19 | (1.12-1.27) | 0.27 | (0.20-0.26) |
|  |  |  |  |  |  |  |
| **Overweight/obesity** | **Term-SGA** | | **Term-AGA** | | **Term-LGA** | |
|  | aOR | 95%CI | aOR | 95%CI | aOR | 95%CI |
| Total effect | 1.54 | (1.40-1.70) | reference | | 0.56 | (0.52-0.60) |
| Direct effect | 0.47 | (0.43-0.51) | reference | | 2.48 | (2.36-2.61) |
| Indirect effect |  |  |  |  |  |  |
| via human milk feeding | 1.00 | (0.99-1.01) | reference | | 1.00 | (1.00-1.00) |
| via human milk feeding rapid weight gain | 3.30 | (3.13-3.46) | reference | | 0.23 | (0.21-0.24) |
|  |  |  |  |  |  |  |
| **Overweight/obesity** | **Post-term-SGA** | | **Post-term-AGA** | | **Post-term-LGA** | |
|  | aOR | 95%CI | aOR | 95%CI | aOR | 95%CI |
| Total effect | 0.93 | (0.63-1.25) | 0.51 | (0.41-0.61) | 0.35 | (0.15-0.54) |
| Direct effect | 0.62 | (0.43-0.80) | 0.92 | (0.77-1.06) | 3.12 | (2.15-4.10) |
| Indirect effect |  |  |  |  |  |  |
| via human milk feeding | 1.00 | (1.00-1.00) | 1.00 | (1.00-1.00) | 1.00 | (0.99-1.00) |
| via human milk feeding rapid weight gain | 1.52 | (1.30-1.73) | 0.56 | (0.50-0.61) | 0.11 | (0.05-0.16) |

Table S. 4.4- Pathway linking size at birth, exclusive human milk feeding, rapid weight to overweight/obesity at least once, direct and indirect effect adjusted for setting and child age

Table S.4.5 The association between size at birth and gestational age with rapid weight gain with human milk feeding and rapid weight gain at 12 months by imputing missing data.

|  | **Exclusive human milk feeding 6 months** | | **Rapid weight gain at 12 months** | |
| --- | --- | --- | --- | --- |
|  | aOR* | 95% CI | aOR* | 95% CI |
| Number children | N=380,406 |  | N=380,406 |  |
| Model 1 |  |  |  |  |
| SGA | 0.83 | (0.81,0.85) | 2.38 | (2.32,2.44) |
| AGA (reference) | 1.00 | -- | 1.00 | -- |
| LGA | 0.95 | (0.93,0.97) | 0.34 | (0.33,0.36) |
|  |  |  |  |  |
| Preterm | 0.45 | (0.44,0.47) | 1.23 | (1.18,1.27) |
| Term (reference) | 1.00 | -- | 1.00 | -- |
| Post-term | 1.16 | (1.11,1.21) | 0.66 | (0.62,0.70) |
| Model 2 |  | |  | |
| Preterm-SGA | 0.27 | (0.24,0.30) | 4.21 | (3.79,4.66) |
| Preterm-AGA | 0.41 | (0.39,0.42) | 1.20 | (1.16,1.25) |
| Preterm-LGA | 0.61 | (0.58,0.65) | 0.36 | (0.33,0.40) |
| Term-SGA | 0.82 | (0.80,0.85) | 2.34 | (2.28,2.40) |
| Term-AGA (reference) | 1.00 | --, | 1.00 | -- |
| Term-LGA | 0.90 | (0.88,0.93) | 0.35 | (0.34,0.37) |
| Post-term-SGA | 1.13 | (1.04,1.24) | 1.39 | (1.26,1.54) |
| Post-term-AGA | 1.09 | (1.03,1.15) | 0.70 | (0.65,0.75) |
| Post-term-LGA | 1.14 | (0.97,1.33) | 0.21 | (0.15,0.30) |
